# A Novel Rehabilitative Protocol in the Treatment of Mixed Urinary Incontinence in Women: The Effects of Focused Mechano-Acoustic Vibration

**DOI:** 10.1089/biores.2019.0041

**Published:** 2019-12-20

**Authors:** Teresa Paolucci, Rosa Grazia Bellomo, Letizia Pezzi, Franco Frondaroli, Serena Frondaroli, Alessandro Santarelli, Claudia Barbato, Annamaria Porreca, Raoul Saggini

**Affiliations:** ^1^ Unit of Physical Medicine and Rehabilitation, Department of Medical and Oral Sciences and Biotechnologies (DSMOB), G. d'Annunzio University of Chieti-Pescara, Chieti, Italy.; ^2^ Department of Biomolecular Science, Physical Medicine and Rehabilitation, Carlo Bo University Study of Urbino, Urbino, Italy.; ^3^ Obstetric-Gynecological Clinic (Maternal-Infant Department), University Hospital of “SS. Annunziata”, Chieti, Italy.; ^4^ Complex Gynecological Operative Unit, “Floraspe Renzetti” Hospital, Lanciano, Italy.; ^5^ Department of Economic Science, G. d'Annunzio University, Chieti-Pescara, Italy.

**Keywords:** exercise, pelvic muscle training, physical therapy, posture, rehabilitation, urinary incontinence

## Abstract

Dysfunctions of the pelvic floor related to mixed urinary incontinence in women are pathologies extremely limiting for patients bodily and psychosocial conditions, altering their quality of life. The aim of this study was to determine the effects of focal mechanical vibrations in mixed urinary incontinence. In this retrospective observational case–control study, 65 patients were randomized and divided into 2 groups: treatment group by focal mechanical vibrations (VISS-10 sessions) (*N* = 33) and a control group in waiting list (*N* = 32). Also, both groups received home-based postural ergonomic instructions to reinforce pelvic floor. Data were collected at T0 (baseline), T1 (end of treatment), and T2 (follow-up = after 1 month): rheological muscle parameters were assessed by MyotonPRO respect to evaluate the gluteus maximus muscle. Then, to measure the general disability of the pelvic floor and the impact of urogenital problems on daily activities the Pelvic Floor Disability Index (PFDI-20) and the Pelvic Floor Impact Questionnaire (PFIQ-7) were used. Groups were matched perfectly before treatment for age (58.20 ± 4.37 vs. 58.73 ± 5.19) and BMI (26.15 ± 2.22 vs. 25.85 ± 2.11); for the two-way ANOVA analysis, a difference in gluteus variables over time and between groups except for GMDR (group *p*-value = 0.60) was showed. The two-way ANOVA shows statistically significant effects of treatment and time for PDFI-20 and PFIQ-7 (*p*-value <0.001). An improvement in incontinence symptoms and quality of life in the PDFI-20 and PFIQ-7 scores were reported and VISS may favor muscles stiffness for exercises by improving the normalization of basal tone. Our results were encouraging and suggested the use of focal mechanical vibration as a novel tool for treating mix urinary incontinence in women to complete and help the rehabilitative therapeutic protocol.

## Introduction

Urinary female incontinence, defined by the International Continence Society as the complaint of any involuntary leakage of urine, is a pathology that has significant implications for patients and their families and a tremendous economic impact on health services.^1,2^ There are various forms of urinary incontinence, each of which is identified specifically with regard to symptoms and causes: stress urinary incontinence (SUI, involuntary leakage on effort or exertion or on sneezing or coughing), urge incontinence (UI, an involuntary leakage that is accompanied or immediately preceded by urgency), mixed incontinence (MI, involuntary leakage that is associated with urgency, exertion, effort, sneezing, or coughing), and incontinence due to regurgitation.^3–5^

However, urinary incontinence is a debilitating problem, because it prevents daily activities and disrupts the psychological well-being of women who are affected, especially those aged 65 years or older.^6^ It is often caused by a weakening of the pelvic floor, blockage of the urethra, or a decrease in the sphincter tone of the urethra, but there are other causes, such as urinary or vaginal infections, secondary effects of certain drugs, muscle weakness, nerve or muscle diseases, and secondary effects of surgical procedures.^7–9^

Urinary incontinence can be treated successfully, often by combining several approaches, such as surgical therapy, pharmacological therapy, and pelvic re-education.^10,11^

Moreover, compared with stress incontinence (SI), women with UI, and MI report significantly higher ratings of urinary urge intensity, more incontinence episodes, and worse in quality of life.^12^ The higher-than-expected prevalence of MI could be due to SI and UI episodes occurring at separate times but being linked by a common predisposing factor.^13,14^ Clinical practice guidelines recommend individualized pelvic floor muscle (PFM) training as a first-line treatment for stress or mixed UI in women, although the lack of personnel and financial resources has limited the delivery of this modality.^11^

Alternatively, or in parallel with any type of surgical or pharmacological intervention, a physical rehabilitation approach, known as pelvic re-education, is based on the possibility of urging and accustoming the patient to self-management of muscular contractions. Muscles, such as the adductor magnus (AM), gluteus maximus (GM), rectus abdominis (RA), and abdominal external and internal oblique muscles, have important functions in the treatment of SUI, and a relationship between the contraction of these muscles and PFMs has been established.^15,16^ In addition, the co-activation of abdominal and PFMs is significant in regulating internal abdominal pressure, which is critical for proper continent function.

The nature of this re-education is above all preventive, but the progress in technology has meant that in certain cases, pelvic rehabilitation can be implemented as auxiliary healing therapy. In rehabilitation, among physical therapies using medical devices, mechanical focal vibration has generated good results with regard to muscle strengthening.^17^ Also, using high-frequency focal vibration, Saggini et al. normalized the basal muscle tone in healthy persons.^18^ No study has examined the use of focal mechanical vibration in the reinforcement and normalization of tone and stiffness in the floor and pelvic girdle muscles that are implicated in mixed urinary incontinence (MUI) incontinence in women.

Based on this premise, the aim of this study was to determine the effects of focal mechanical vibrations in MUI. The primary outcome was the improvement in symptoms per the Pelvic Floor Disability Index (PFDI-20),^19^ and the secondary outcome was the evaluation of tone and stiffness of the GM muscle.

## Materials and Methods

This retrospective observational case–control study examined the effects of focused mechano-acoustic vibration therapy (VISS) in the rehabilitation of MUI in women per the Strobe Guidelines.^20^

Seventy-six (*N* = 76) patients with MUI were recruited from the gynecology outpatient clinic of G. D'Annunzio University of Chieti-Pescara; 74 patients agreed to participate, and 65 met the inclusion criteria and were enrolled, visiting the physical medicine and rehabilitation outpatient clinic of this institution from June 2018 to January 2019.

Sample randomization (1:1) was performed, and the patients were divided into two groups: treatment with VISS (EX) (*N* = 33) and a wait list (CT) (*N* = 32).

The inclusion criteria were age between 50 and 70 years; a diagnosis of MUI, as defined by the International Continence Society (ICS)^4^; and body mass index (BMI) <30. The exclusion criteria were participation in other therapeutic protocols and the presence of urogenital infections, neurological pathologies, pelvic organ prolapse, pain, hematuria, voiding symptoms, and spinal lesions of any grade. Women with prior anti-incontinence surgery or medical treatment for overactive bladder were excluded. The clinician (a specialist in gynecology) assessed the predominant type of incontinence and its duration and performed a urine analysis and urine culture. The physical examination included abdominal, pelvic, and vaginal examinations and a stress test.

This study was performed per the Helsinki Declaration on human experimentation and was approved by the Departmental committee of G. D'Annunzio University of Chieti (Italy). All patients gave written informed consent after receiving detailed information on the study's aims and procedures. Patients were evaluated at T0 (before the start the protocol), T1 (after completion of the rehabilitative protocol), and T2 (1-month follow-up).

In addition, patients were asked to report and record any adverse effects during the rehabilitative treatment, daytime and night-time losses, and intervals that were free from urine leakage (hours), quantifying urine loss (drops per day) in an individual voiding diary.

### Outcome measures

To measure rheological muscle parameters, a MyotonPRO^21–23^ was used to evaluate the GM.^15^ Specifically, the following parameters were considered: muscular tone (F-oscillation frequency), to indicate the basal involuntary mechanic tension of the muscle, expressed in Hertz (Hz); muscular elasticity/plasticity (D-logarithmic decrement), as the capacity of a muscle to recover its initial shape after mechanical modification; and muscular resistance (S-dynamic stiffness), as the property of the muscle to counteract external mechanical deformation through the activation of antagonist muscles (this value correlates with muscular tone and is expressed in N/m).

These measurements were made by applying the needle-shaped pressure sensor of the instrument to the center of the selected muscle belly. The instrument, after repeating 3 percussions on the muscle, yielded values regarding its tone, elasticity, and stiffness, also calculating the probability of error of the measurement. The accepted probability of error was set to 2%.

The PFDI-20 measures the general disability level of the pelvic floor, considering symptoms over the past 3 months before the evaluation. It is composed of three subscales:POPDI-6, CRADI-8, and UDI-6. The score for each item varies from 0 (no symptoms) to 4 points (extremely disabling symptoms). For each scale, the average score of the answers is calculated (ranging from 0 to 4 points) and multiplied by 25 (100/4) to obtain the final score of the subscale (from 0 to 100). The final PFDI-20 score is derived from the sum of each subscale (between 0 and 300 points).^19^

The Pelvic Floor Impact Questionnaire (PFIQ-7) determines the impact of urogenital problems on daily activities, considering symptoms from the 3 months before evaluation. It comprises seven items, each of which is related to a specific urogenital symptom. The score for each item varies from 0 (absent) to 3 points (extremely present). Each symptom is assessed regarding its presence at the level of the bladder/urine (UIQ-7), intestine/rectus (CRAIQ-7), and vagina/pelvis (POPIQ-7). For each subscale, the mean score is calculated (from 0 to 3 points) and multiplied by 33.3 (100/3) to obtain the final score for the subscale (0–100 points). The final PFIQ-7 score is derived from the sum of each subscale (0–300 points).^24^

### Rehabilitative treatment group

**FIG. 1.**
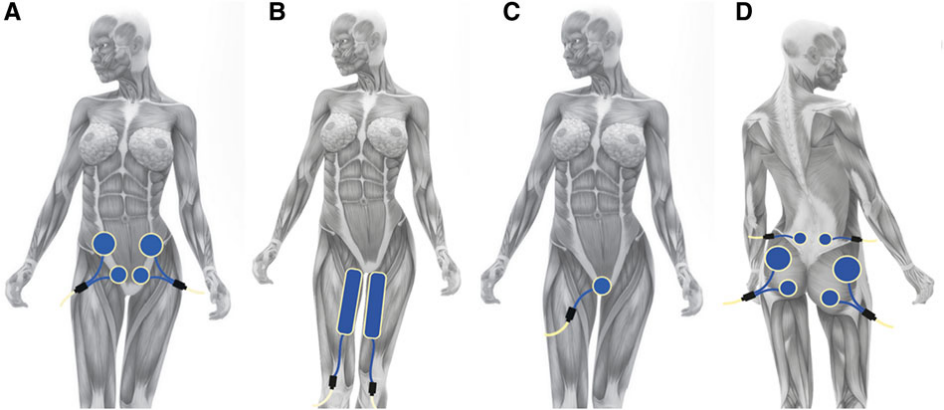
**(A–D)** The anatomic model shows the positioning of the transducers by VISS with respect to the aforementioned reference muscles. **(A)** abdominal muscles; **(B)** adductors muscles; **(C)** pelvic floor muscle; **(D)** gluteus maximus muscle and quadratus lumborum muscles.

The experimental therapeutic protocol (EX), consisted of 10 sessions, 3 times per week for the first 2 weeks and then twice weekly for the next 2 weeks. A frequency of 300 Hz for 15 min was used for every muscle that was treated by the expert physiotherapist during the treatment. Finally, a follow-up period of 1 month ensued without treatment. Each patient underwent focused mechano-acoustic vibration with applicators placed bilaterally at the level of the rectus abdominis, adductor muscles (gracilis, pectineus, long adductor, and short adductor muscles), GM, quadratus lumborum, and perineal area (Fig. 1).

**FIG. 2.**
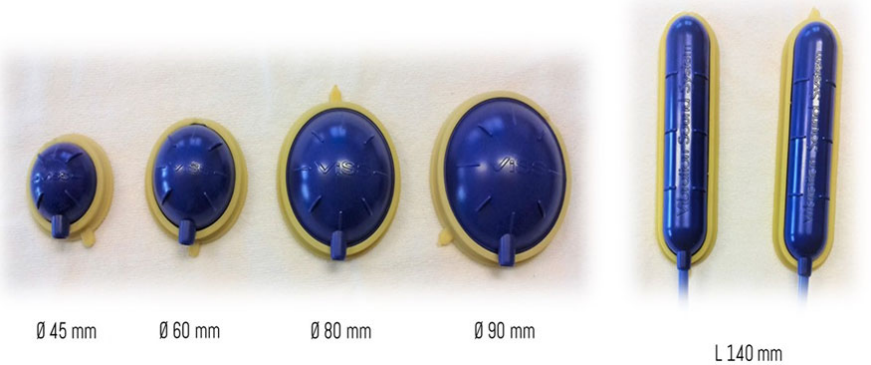
VISS transducers. The transducers are of different shape and size; they are composed of ABS, a common thermoplastic polymer with mechanical properties as impact and heat resistance and toughness, while the lateral bearing is made of Santoprene TPV. ABS, acrylonitrile butadiene styrene.

The focused mechano-acoustic vibration was administered using the Vibration Sound System^®^ *(European patent: Ep1824439–CE 1936 Certificate of Conformity - N° HD 60114019 - Unibell, Calco - LC, Italy)* (Fig. 2). This instrument consists of a 32,000-revolution turbine with a flow rate of 35 m^3^/h that generate air waves with a pressure of up to 250 mbar and a flow modulator that vibrates air with a pressure of up to 630 mbar and frequency of up to 980 Hz (however, a frequency up to 300 Hz is recommended) for producing mechano-acoustic waves.^25^

**FIG. 3.**
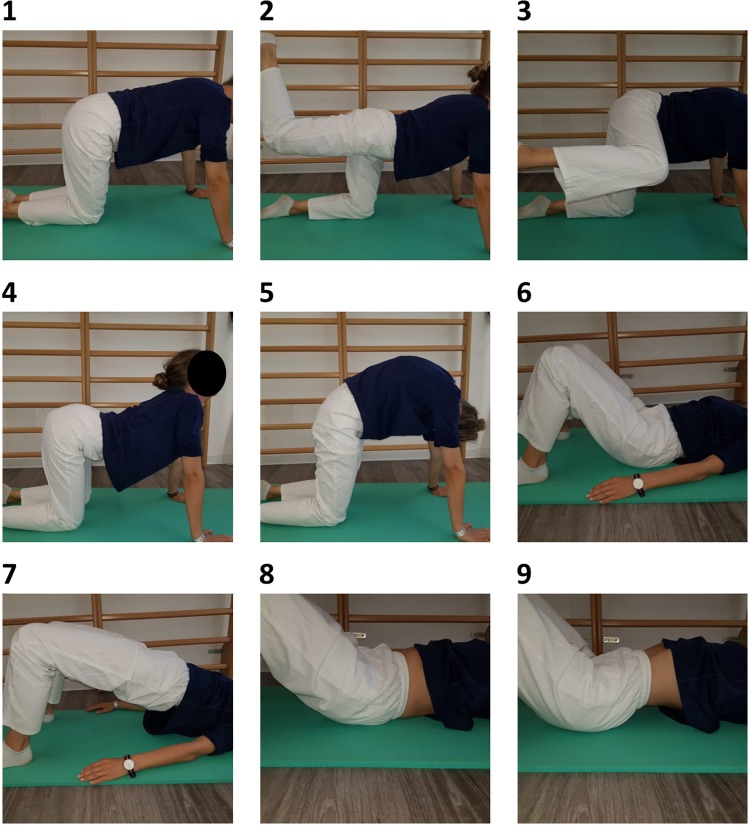
Home-made exercises. Exercises 1-2-3 = exercises to strengthen gluteus muscles (hip kicker); Exercises 4–5 = exercises to strengthen upper and lower back muscles (angry cat stretch); Exercises 6 = abdominal breathing; Exercises 6–7 = exercises to strengthen gluteus muscles and pelvic floor (basic bridge); Exercises 8–9 = exercises to strengthen pelvic floor (pelvic tilt).

Whole patients as in the EX and CT (waiting list) received postural ergonomic instructions that were to be put into practice in everyday life to improve urinary incontinence symptoms; trained with respect to isometric contraction exercises for the GM, tilting exercises for the pelvis, and diaphragmatic breathing exercises; and instructed to walk at least 120 min per week. Every patient was given a pamphlet to explain the home exercises (Fig. 3).

### Statistical evaluation

#### Sample size calculation

The following conditions were set to determine the sample size: a significance level of 95% and a power of 80% under the hypothesis of an average variation in PFDI-20 scores^19^ of 60.00 ± 19.19 for EX (VISS) versus CT (wait list) after treatment. As a result, 30 patients for each group were enrolled, considering a dropout rate of 20%.

#### Data analysis

Data were collected at T0, T1, and T2 to determine how the patients, regardless of treatment, improved their conditions. Differences in weight, age, BMI, and height were analyzed by *t*-test. The normality of the data distribution was examined by Jarque-Bera test, and covariance sphericity was analyzed by Mauchly test- Differences in the mean values for the gluteus maximum (right and left sides- R and L) variables (muscular tone-F, muscular elasticity/plasticity-D, and muscular resistance-S) in each group over time were assessed by two-way ANOVA for repeated measurements, followed by Tukey's *post hoc* test: GMD = gluteus maximus muscle logarithmic decrement; GMF = gluteus maximus muscle oscillation frequency [Hz]; GMS = gluteus maximus muscle dynamic stiffness [N/m].

Except for oscillation frequency right (GMFR) for the gluteus maximus, the variables violated the normality and sphericity assumptions; thus, these data were subjected to Tukey's ladder of powers transformation. Further, the PFDI-20 and PFIQ-7 scales were tested by repeated measures two-way ANOVA.

Before parametric analysis was performed, descriptive statistics were used to describe the sample. Differences were evaluated at *p*-value <0.05. All analyses were performed in R environment, and the sample size was calculated with G-power, version 3.1.

## Results

**FIG. 4.**
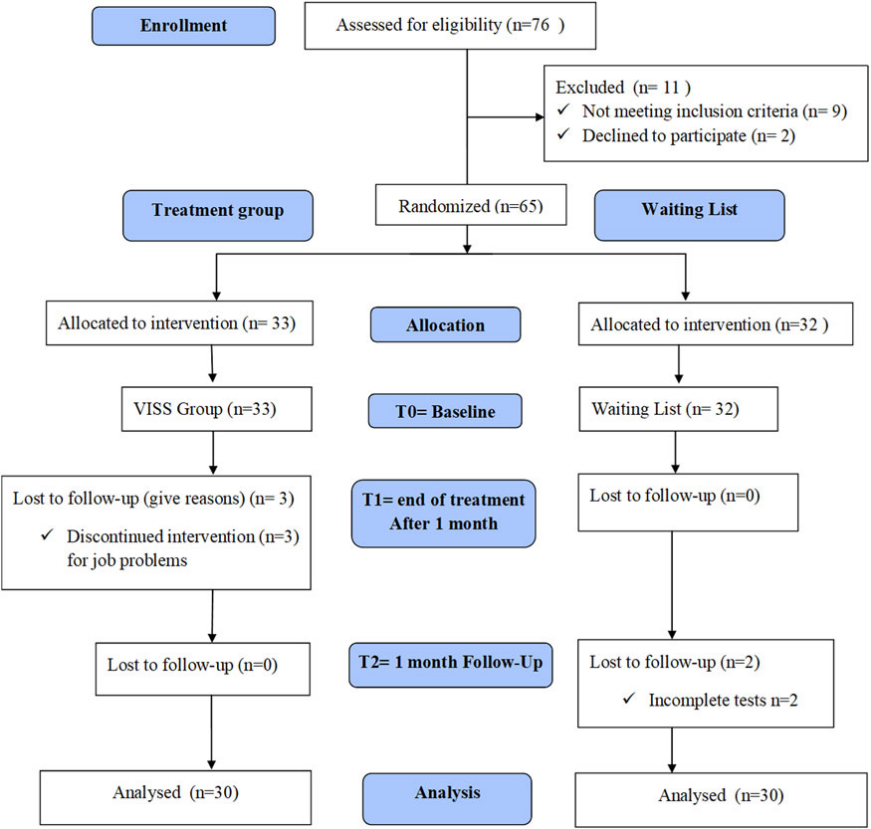
Flowchart.

The data of the 60 patients were calculated (Fig. 4). Tables 1 and 2 lists the descriptive statistics for each variable (clinical scales and MyotonPro values) in the two groups. Groups were matched perfectly before treatment for age and BMI, and the means for the groups did not differ statistically at baseline: the mean age of the EX was 58.20 ± 4.37 years versus 58.73 ± 5.19 years in the CT; for mean BMI (kg/m^2^) in the EX and CT was 26.15 ± 2.22 and 25.85 ± 2.11, respectively.

**Table 1.** Mean and SD for Every Variable in the Control (CT) and Treatment (EX) Groups

Control group (CT)		Treatment group (EX)
Variable	Mean ± SD	Mean ± SD
Age (years)	58.20 ± 4.37	58.73 ± 5.19
Weight (kg)	72.23 ± 9.03	71.47 ± 9.15
Hight (m)	1.66 ± 0.06	1.66 ± 0.06
BMI (kg/m^2^)	26.15 ± 2.22	25.85 ± 2.11

**Table 2.** Mean and SD for Outcome Measures in the Control (CT) and Treatment (EX) Groups

Variable	T0		T1		T2	
	EX	CT	EX	CT	EX	CT
GMDL	1.60 ± 0.28	1.55 ± 0.28	1.74 ± 0.28	1.68 ± 0.34	1.77 ± 0.28	1.69 ± 0.35
GMDR	1.57 ± 0.27	1.60 ± 0.29	1.74 ± 0.29	1.71 ± 0.34	1.77 ± 0.30	1.72 ± 0.34
GMFL (Hz)	13.02 ± 1.13	13.15 ± 1.46	11.77 ± 1.53	12.39 ± 1.69	11.76 ± 1.57	12.49 ± 1.75
GMFR (Hz)	13.02 ± 1.04	13.17 ± 1.2	11.68 ± 1.30	12.25 ± 1.72	11.73 ± 1.40	12.32 ± 1.74
GMSL (N/m)	251.11 ± 24.56	307.81 ± 20.53	226.77 ± 20.24	300.23 ± 19.99	230.01 ± 19.2	300.40 ± 19.67
GMSR (N/m)	222.39 ± 18.10	226.59 ± 22.11	212.5 ± 16.19	220.83 ± 22.08	213.16 ± 15.53	221.62 ± 21.48
PFDI-20 (0–300)	77.13 ± 21.17	77.30 ± 23.74	49.20 ± 17.18	79.20 ± 21.11	53.83 ± 19.22	77.43 ± 21.74
PFIQ-7 (0–300)	110.93 ± 24.88	109.60 ± 27.13	68.17 ± 24.14	110.93 ± 24.88	71.27 ± 21.74	117.63 ± 30.17

GMDL, gluteus maximus muscle logarithmic decrement Left; GMDR, gluteus maximus muscle logarithmic decrement Right; GMFL, gluteus maximus muscle oscillation frequency Left; GMFR, gluteus maximus muscle oscillation frequency Right; GMSL, gluteus maximus muscle dynamic stiffness Left; GMSR, gluteus maximus muscle dynamic stiffness Right; PFDI-20, Pelvic Floor Disability Index; PFIQ-7, Pelvic Floor Impact Questionnaire.

**FIG. 5.**
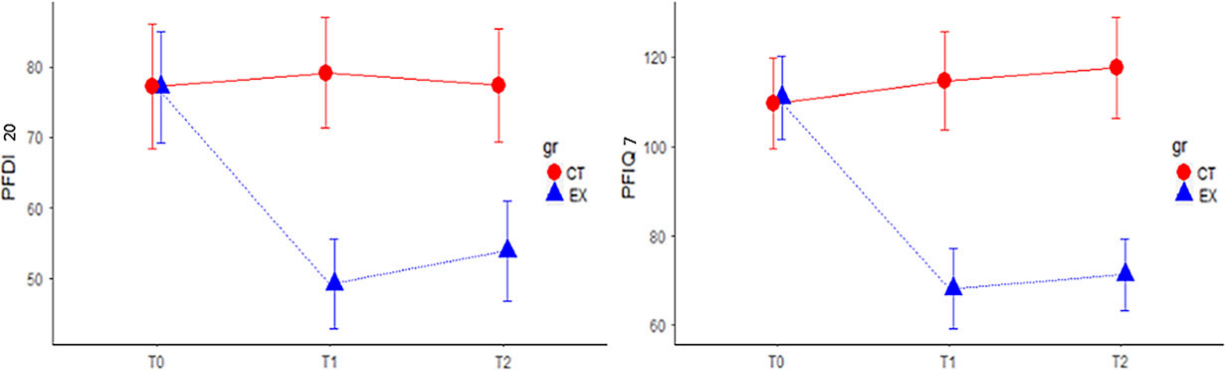
Interaction plot of PDFI-20 and PFIQ-7 variables at three time points for the control (CT) and treatment groups (EX). PFDI-20, pelvic floor disability index; PFIQ-7, pelvic floor impact questionnaire; T0 = at baseline; T1 = at the end of treatment; T2 = at follow-up.

Table 3 shows the results of the two-way ANOVA, confirming the differences in gluteus variables over time and between groups except for GMDR (group *p*-value = 0.60). The principal effects were statistically significant, but their interaction was not. Thus, for every variable, except GMDR, being treated or not had a different effect on the outcome over time. Table 4 shows the *p*-values and effect sizes for PDFI-20 and PFIQ-7, which were statistically significant with regard to the principal effects and their interaction, indicating that there were significant differences between groups and over time. Thus, the change in variables over time differed, depending on group membership. Figure 5 shows the trends of PDFI-20 and PFIQ-7.

**Table 3.** Two-Way ANOVA of Gluteus Muscle Variables

Factors	GMDL	GMDR	GMFL	GMFR	GMSL	GMSR
*p*-values (Effect size)						
Group	<0.05 (0.023)	0.60 (0.002)	<0.05 (0.023)	<0.05 (0.021)	<0.001 (0.698)	<0.05 (0.029)
Time	<0.01 (0.057)	<0.01 (0.055)	<0.001 (0.095)	<0.001 (0.118)	<0.001 (0.030)	<0.05 (0.044)
Interact.	0.89 (0.001)	0.71 (0.004)	0.44 (0.008)	0.63 (0.004)	0.19 (0.005)	0.63 (0.005)

GMDL, gluteus maximus muscle logarithmic decrement Left; GMDR, gluteus maximus muscle logarithmic decrement Right; GMFL, gluteus maximus muscle oscillation frequency Left; GMFR, gluteus maximus muscle oscillation frequency Right; GMSL, gluteus maximus muscle dynamic stiffness Left; GMSR, gluteus maximus muscle dynamic stiffness Right.

**Table 4.** Two-Way ANOVA of PDFI-20 and PFIQ-7

Factors	PFDI-20	PFIQ-7
*p*-values (Effect size)		
Group	<0.001 (0.0142)	<0.001 (0.041)
Time	<0.001 (0.062)	<0.001 (0.063)
Interact.	<0.001 (0.078)	<0.001 (0.079)

PFDI-20, pelvic floor disability index; PFIQ-7, pelvic floor impact questionnaire.

No adverse effects were reported during the rehabilitative treatment.

## Discussion

The main finding of this study was the positive effect of VISS on reducing disability index scores with respect to MUI in the EX versus CT. In particular, in the VISS group, patients reported improvements in their urinary incontinence symptoms and quality of life, their PDFI-20 and PFIQ-7 scores were statistically significant, and the change over the three evaluation times differed, depending on group membership.

The patients' perception of their disability with regard to MUI became positive, the EX group received intensive treatment, at least in the first 2 weeks, likely ensuring greater compliance with the rehabilitation with VISS continuity with the home-based exercises. The association between VISS and exercises that reinforce the GM muscle, tilting exercises for the pelvis, diaphragmatic breathing exercises, and instructions to walk at least 120 min per week showed encouraging positive results in improving MUI. Compared with previous studies,^25,26^ mechanical focal vibration can be a good approach for rehabilitating the PFMs; it is also simpler and less invasive than other techniques used.

VISS is a new approach to MUI, the mechanism of action on the PFMs and other muscles that could be attributed to the stimulation of mechanoreceptors, in particular the Pacinian corpuscles, which have the highest sensitivity (1 mm) at a frequency of 250–300 Hz, thus representing the target vibration receptors,^27^ and the consequent muscle training due to a frequency of 300 Hz, as in our rehabilitation protocol.^28,29^

Also, other groups have reported that high-frequency focal vibration induces long-term reorganization of the primary motor cortex,^17,30–32^ characterized by persistent increases in intracortical and cortical reciprocal inhibition, which might reduce undesired muscle activation and minimize co-contractions, consequently enhancing motor performance. We hypothesize that this mechanism would allow better integration between the PFMs and pelvis-stabilizing muscles, such as the rectus abdominis, in controlling the posterior tilting of the pelvis, GM, and large adductor, such as synergistic muscles of the rectus abdominis. During the Valsalva maneuver, incontinent women experience greater anteroposterior displacement than continent women, and the deep layer of the PFM is more tightly modulated by respiration than the superficial layer, but activation of the superficial layer was extensive during maximal/submaximal occluded respiratory efforts and earlier during cough.^33,34^

Thus, in our rehabilitative protocol, importance was given to teaching and adopting diaphragmatic breathing in home-based exercises. Also, such muscles as the AM, GM, RA, and abdominal external and internal oblique muscles, are critical in the treatment of urinary incontinence: synergistic activation of these muscles intensifies a woman's ability to contract the PFM.^15^

The objectives of our rehabilitative intervention were to promote awareness of the contraction of the pelvic muscles in patients; control and, if possible, eliminate the synergies between agonist and antagonist muscles that are involved in the biomechanics of the pelvic floor; and integrate the rehabilitative program with activities of daily living by promoting walking for at least 120 min per week.

Our results showed normalization of basal tone (F), plasticity (D), and stiffness (S) in the EX and CT with respect to the GM, which was used as the reference in the MyotonPro analysis.^35^ Thus, it is not possible to conclude that VISS is better than the home-based exercises that were proposed to the patients. Nevertheless, the EX experienced major changes in rheological properties of the muscle with versus the CT, except for GMDR (group *p* = 0.60). We assume that VISS prepares muscles for home-based exercises by improving the normalization of basal tone.

The lack of a time-group interaction could be due to various factors: it was difficult to find homogeneity of the rheological parameters of the muscles at T0, which rendered the two groups perfectly comparable, and moreover, the MytonPro is useful in clinical studies, but it likely has lower sensitivity than surface electromyography, due to a bias that is linked to the repeatability of the measurements.^15^ Compared with the clinical scales, patients with MUI episodes in the EX experienced significantly greater improvement versus the CT; the combination of VISS and home-based exercises could have effected better functional motor coordination of the PFMs and agonist muscles that were treated with focal mechanical vibration. A strength of this study is that it is the first attempt to use VISS for MUI. One weakness was the lack of a control group of healthy female participants for evaluating and comparing the rheological properties of the GM by MyotonPro analysis.

## Conclusion

In conclusion, our results are encouraging and suggest the use of VISS by high-frequency focal vibration as a valid therapeutic tool for treating MUI in women. The prescription of simple home-based exercises for pelvic tilting and diaphragmatic breathing, which are involved in the control of urinary function and intra-abdominal pressure, will allow the patient to become aware of the contraction of the PFMs.

## Abbreviations Used

AM: adductor magnus
BMI: body mass index
GM: gluteus maximus
GMD: gluteus maximus muscle logarithmic decrement
GMF: gluteus maximus muscle oscillation frequency
GMS: gluteus maximus muscle dynamic stiffness
MI: mixed incontinence
MUI: mixed urinary incontinence
PFDI: pelvic floor disability index
PFIQ: pelvic floor impact questionnaire
PFM: pelvic floor muscle
RA: rectus abdominis
SI: stress incontinence
SUI: stress urinary incontinence
UI: urge incontinence
